# Supplementary material for: Exploring the Clinical Utility of the Music Therapy Assessment Tool for Awareness in Disorders of Consciousness (MATADOC) with People with End-Stage Dementia
Source: Brain Sci. 2022 Sep 28;12(10):1306. doi: 10.3390/brainsci12101306 (PMC9599261; doi:10.3390/brainsci12101306)
Supplement: Supplementary file 1 [file brainsci-12-01306-s001.zip › brainsci-1910615-supplementary.pdf]

Clinician evaluation of MATADOC clinical utility for people with end-stage dementia

Therapist initials:_________ **Patient identifier (for your purpose only):___________________** **Date:________________**

Your evaluation of the MATADOC is ***invaluable***. Please think about this patient over the four sessions.

Select an answer (where relevant) by circling it. Add comments where you would like to.

**EVALUATION OF THE CLINICAL PROTOCOL FOR PEOPLE WITH END-STAGE DEMENTIA**				
**Your evaluation of the observation period for these clients**				COMMENTS (e.g. What could have been different?)
In your opinion, was the observation period too short, too long, just right?	Too long	Too short	Just right	
**YOUR EVALUATION OF THE *TREATMENT PROTOCOL* FOR CLIENTS WITH END-STAGE DEMENTIA**				
**Introduction of musical stimulus procedure**				COMMENTS (e.g. What could have been different?)
Thinking about this patient, did you feel this procedure was ***appropriate*** for this patient?	Yes	No	NA	
What could have been different from the protocol directions? E.g. music more stimulating; singing more words; use of instrument.				
**Presentation of auditory stimuli procedure (i.e. playing instruments to one side)**				COMMENTS (e.g. What could have been different?)
Thinking about this patient, did you feel this procedure was ***appropriate*** for this patient?	Yes	No	NA	
Thinking about this patient, did you feel this procedure was ***relevant***?	Yes	No	NA	
Did the auditory stimuli procedure provide new information about this patient?	Yes	No	Other	
**Visual stimuli procedure (i.e. moving an instrument in the client's visual gaze):**				COMMENTS (e.g. What could have been different?)
Thinking about this patient, did you feel this procedure was ***appropriate*** for this patient?	Yes	No	NA	
Thinking about this patient, did you feel this procedure was ***relevant***?	Yes	No	NA	
Did the visual stimuli procedure provide new information about this patient?	Yes	No	Other	
**Verbal command procedure:**				COMMENTS (e.g. What could have been different?)
Thinking about this patient, did you feel this procedure was ***appropriate*** for this patient?	Yes	No	NA	
Thinking about this patient, did you feel this procedure was ***relevant***?	Yes	No	NA	
Did the verbal command procedure provide new information for you?	Yes	No	Other	
What verbal commands worked best for this patient?				

**Familiar song procedure**				COMMENTS
Thinking about this patient, did you feel this procedure was ***appropriate*** for this patient?	Yes	No	NA	
Thinking about this patient, did you feel this procedure was ***relevant***?	Yes	No	NA	
Thinking about this patient, **does the MATADOC protocol *overall***				
Fit your usual working practices?	Yes	No	NA	
Challenge your usual working practices?	Yes	No	NA	
Improve your usual working practices?	Yes	No	NA	
In your opinion, are there procedures that should be added for people with end-stage dementia?				
In your opinion overall, did the MATADOC ***provide new information*** about this patient?	Yes	No	NA	
In your opinion overall, was the MATADOC ***useful*** in music therapy care for this patient?	Yes	No	NA	
Did the MATADOC require ***special arrangements for selecting / altering environments?***	Yes	No	NA	

YOUR EVALUATION OF THE MATADOC *ITEMS* FOR CLIENTS WITH END-STAGE DEMENTIA (please mark/circle one for relevance AND usefulness where you can)

**Item 1: Responses to visual stimuli**	Very relevant	Somewhat relevant	Neutral	Not really relevant	Not relevant	Very useful	Somewhat useful	Neutral	Not really useful	Not useful
**Item 2: Responses to auditory stimuli**	Very relevant	Somewhat relevant	Neutral	Not really relevant	Not relevant	Very useful	Somewhat useful	Neutral	Not really useful	Not useful
**Item 3: Awareness to musical stimuli**	Very relevant	Somewhat relevant	Neutral	Not really relevant	Not relevant	Very useful	Somewhat useful	Neutral	Not really useful	Not useful
**Item 4: Verbal commands**	Very relevant	Somewhat relevant	Neutral	Not really relevant	Not relevant	Very useful	Somewhat useful	Neutral	Not really useful	Not useful
**Item 5: Arousal**	Very relevant	Somewhat relevant	Neutral	Not really relevant	Not relevant	Very useful	Somewhat useful	Neutral	Not really useful	Not useful
**Item 6: Behavioural response to music**	Very relevant	Somewhat relevant	Neutral	Not really relevant	Not relevant	Very useful	Somewhat useful	Neutral	Not really useful	Not useful
**Item 7: Musical response**	Very relevant	Somewhat relevant	Neutral	Not really relevant	Not relevant	Very useful	Somewhat useful	Neutral	Not really useful	Not useful
**Item 8: Vocalisation**	Very relevant	Somewhat relevant	Neutral	Not really relevant	Not relevant	Very useful	Somewhat useful	Neutral	Not really useful	Not useful
**Item 9: Non-verbal communication**	Very relevant	Somewhat relevant	Neutral	Not really relevant	Not relevant	Very useful	Somewhat useful	Neutral	Not really useful	Not useful
**Item 10: Choice-making**	Very relevant	Somewhat relevant	Neutral	Not really relevant	Not relevant	Very useful	Somewhat useful	Neutral	Not really useful	Not useful
**Item 11: Motor skills**	Very relevant	Somewhat relevant	Neutral	Not really relevant	Not relevant	Very useful	Somewhat useful	Neutral	Not really useful	Not useful
**Item 12: Attention to task**	Very relevant	Somewhat relevant	Neutral	Not really relevant	Not relevant	Very useful	Somewhat useful	Neutral	Not really useful	Not useful
**Item 13: Intentional behaviour**	Very relevant	Somewhat relevant	Neutral	Not really relevant	Not relevant	Very useful	Somewhat useful	Neutral	Not really useful	Not useful
**Item 14: Emotional response**	Very relevant	Somewhat relevant	Neutral	Not really relevant	Not relevant	Very useful	Somewhat useful	Neutral	Not really useful	Not useful

Are there any ***items*** on the documentation you would like to see added for end-stage dementia, or removed?

ANY OTHER COMMENTS YOU WOULD LIKE TO MAKE? (PLEASE THINK ABOUT THE PROTOCOL AND THE DOCUMENTATION OVERALL IN RELATION TO THIS PATIENT)

Figure S1: Clinical Utility Evaluation modelled on Smart's (2006) Multidimensional Model for Clinician Judgements. See: Smart, A. A multi-dimensional model of clinical utility. *Int. J. Qual. Health Care* **2006,** *18*(5), 377-382.
